# Tracheoesophageal Fistula: Airway Management and Temporization in a Community Hospital Setting

**DOI:** 10.7759/cureus.35838

**Published:** 2023-03-06

**Authors:** Elliott J Chiartas, Jeffery Neurock, Kasia Rubin

**Affiliations:** 1 Anesthesiology, Cleveland Clinic South Pointe Hospital, Cleveland, USA

**Keywords:** tracheoesophageal fistula, advanced airway management, critical care anesthesiology, lung isolation, tef, airway anomalies, difficulty ventilating, critical care

## Abstract

A tracheoesophageal fistula (TEF) is a rare anatomical abnormality that can present significant challenges for the anesthesia provider. TEFs, depending on location and size, can result in aspiration, hypoxia, and difficulty with ventilation in the intensive-care unit (ICU) and operating room (OR) settings. Though usually seen and most commonly described as a congenital abnormality, it can also be an acquired condition in adults. Early recognition and diagnosis of TEF are of paramount importance to avoid respiratory complications. The rapid isolation of the TEF is key to management and different methods can be used to temporize the clinical situation until definitive surgical or endoscopic procedures can be accomplished. We discuss methods of temporization of the clinical situation, especially in a community hospital setting with limited access to immediate and sophisticated treatment.

## Introduction

Tracheoesophageal fistulas (TEFs) are an abnormal connection between the airway and gastrointestinal tract (usually between the esophagus and trachea) that can form in utero during development or can be acquired as an adult. More than 50% of acquired cases are secondary to malignancy but they can also be caused by trauma, infection, prior surgeries to the area, and stents [1]. Esophageal carcinoma is the most likely culprit accounting for 77% of cases, with the incidence of a TEF being 4.5% in patients with primary esophageal cancer [2]. Other causes of an acquired TEF not secondary to malignancy are more commonly seen in critically ill patients that have either had tracheostomy placement or prolonged intubation with the cuff of the endotracheal tube causing trauma. Diagnosis may be made by chest radiography, barium swallow studies, and endoscopic procedures [2].

Median survival following a TEF diagnosis is less than three months [3]. The main cause of morbidity and mortality is secondary to pulmonary complications due to aspiration of gastric contents. Symptoms vary depending on location, rate of formation, and patient comorbidities and may include coughing, coughing after swallowing, recurrent pulmonary infection, and unexplained weight loss. Aspiration may lead to pneumonia and diffuse intrapulmonary damage that makes oxygenation difficult. Mandatory ventilation in the intubated patient may be compromised depending on the location of the anatomical connection between the airway and esophagus. We describe the airway management of a patient with an undiagnosed TEF presenting with respiratory distress.

## Case presentation

**Figure 1:**
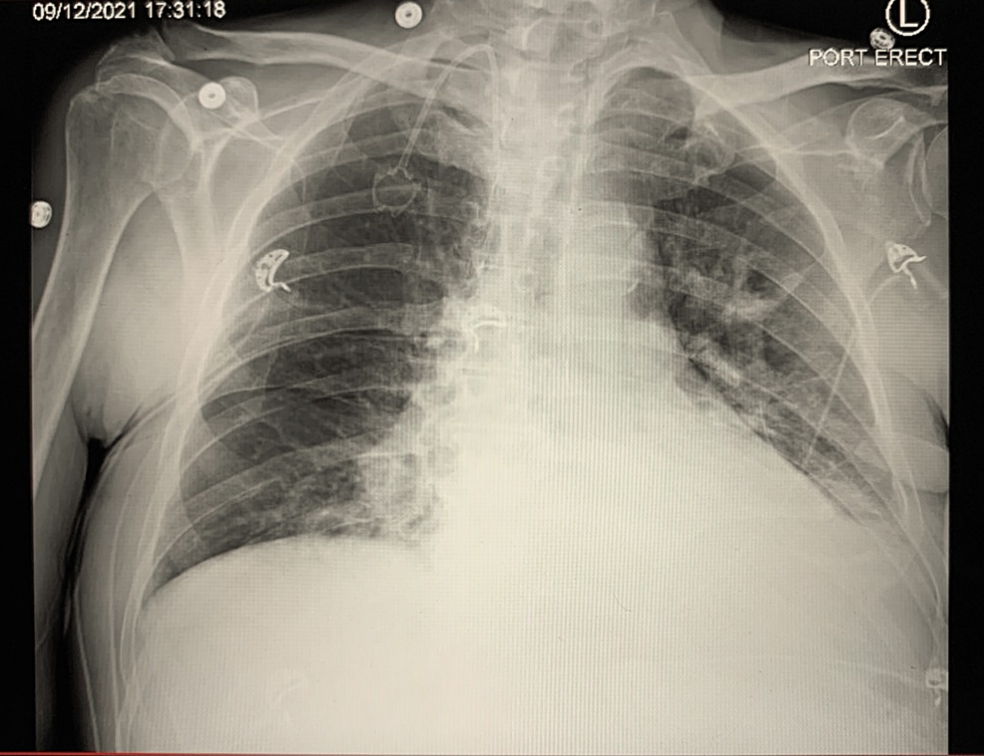
Chest X-ray showing left lower lobe infiltrates on day two of admission

We present a 68-year-old male with known metastatic esophageal carcinoma complicated by radiation-induced pericarditis and a past medical history of hypertension, atrial fibrillation on apixaban (a factor Xa inhibitor), and stable coronary artery disease treated with stents. Prior to admission to our hospital, the patient had been treated at an affiliated quaternary care institution for chest pain and pericardial drain placement secondary to a pericardial effusion. After an extended hospital stay and appropriate treatment, the patient was then discharged to a skilled nursing facility, but the facility was unable to obtain the patient's necessary medications, resulting in admission to a local community hospital. He was admitted in stable condition under the diagnosis of failure to thrive to await placement in a skilled nursing facility. On day two of the current admission, the patient developed a productive cough and complained of worsening shortness of breath, which was managed with nebulizer treatments and minimal supplemental oxygen via nasal cannula. A chest X-ray obtained demonstrated an alveolar infiltrate of the left lower lobe (Figure 1).

On hospital day three, the patient had a large episode of emesis resulting in abrupt hypoxia with oxygen saturations dropping to the low 80s and respiratory distress. At this time, the patient developed sinus tachycardia with rates in the 120s and was hemodynamically stable. The patient's hypoxia did not resolve with supplemental oxygen delivered via a non-rebreather face mask, and he was promptly transferred to the intensive care unit (ICU). Upon arrival at the ICU, his trachea was intubated with a 7.5 endotracheal tube (ETT) via video laryngoscopy by the ICU team using standard doses of rocuronium and etomidate for induction. Immediately upon tracheal intubation, he developed a pulseless wide-complex ventricular tachycardia requiring chest compressions. Return of spontaneous circulation (ROSC) was achieved after roughly two to four minutes of chest compressions and 300 mg of amiodarone.

**Figure 2:**
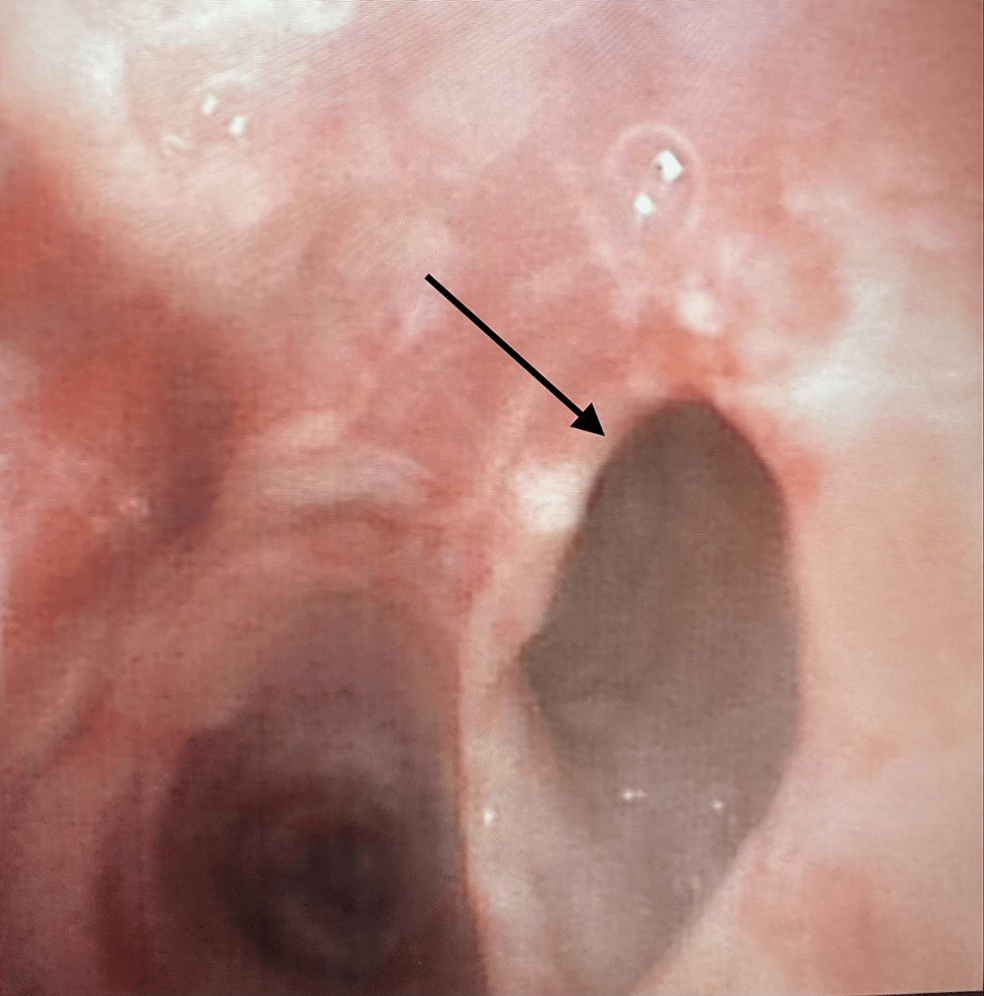
Picture taken from a bronchoscope at the level of the carina with the left mainstem bronchus on the left and the tracheoesophageal fistula on the right

Mandatory intermittent mechanical ventilation using a pressure-controlled, volume-guaranteed mode was initiated following ROSC. The ICU team noted that low-pressure alarms were sounding and that the patient was only receiving 50% of the set tidal volume, with persistent oxygen desaturation in the mid to high 60s. An endotracheal cuff leak was presumed, and the tube was exchanged for an 8.0 ETT with visualization of correct placement using a GlideScope® (Verathon Inc., Bothell, WA). Manual ventilation with cricoid pressure improved patient oxygen saturation to the mid-80s, though an audible leak from the patient’s mouth was still present, and bubbles were noted to come from the esophageal opening upon second visualization of the tracheal tube passing through the vocal cords. A flexible bronchoscope was inserted into the ETT to observe the trachea, bronchial tree, and integrity of the endotracheal tube. A large TEF was seen at the junction of the carina and the left main-stem bronchus (Figure 2). No right-sided double-lumen endotracheal tubes were available at the community hospital, so it was decided that a single-lumen endotracheal tube could be temporarily inserted into the right main-stem bronchus until definitive treatment could be obtained. The single-lumen 8.0 ETT was replaced with an 8.5 ETT, inserted over the fiberoptic bronchoscope, and guided into the right mainstem bronchus. Appropriate placement resulted in an improvement in the patient’s oxygen saturation to the high 80s-low 90s. 

Gastroenterology consultants immediately decided that a stent procedure was not feasible at the community hospital, nor was the patient a good candidate in his current clinical state. It was also determined that the patient was too unstable to be transferred to a quaternary care center where sophisticated resources were available. Due to the patient’s poor prognosis, a goals-of-care discussion with physicians and the patient's spouse determined that further medical intervention was futile. His code status was converted to DNR-Comfort Care, and the patient demised a few hours later.

## Discussion

A low-pressure, or low tidal volume, alarm requires an evaluation of the entire breathing circuit. A breathing system leak due to a ruptured endotracheal tube cuff is a common cause. If an air leak in the ventilator circuit continues despite checking for disconnections and in the presence of a well-inflated cuff, a TEF should be suspected [3]. Diagnosis can be made by flexible bronchoscopy, thoracic imaging studies, and upper endoscopy.

Airway management goals in a patient with TEF aim to improve the current clinical status and to prevent repeated or extensive soiling of pulmonary tissue as aspiration and pulmonary sepsis are the leading causes of mortality [4]. Clinical management techniques to isolate the TEF include fiberoptic bronchoscopy to identify the size and location of the fistula opening, the use of double-lumen tubes to isolate lung ventilation, and spontaneous ventilator strategies. The size and location of the fistula opening determine the method of airway management used. A double-lumen endotracheal tube allows for ventilation of the unaffected lung and provides access for suction through the tracheal lumen. This often aids in an improvement of the patient's clinical status and is the preferred airway management method when the fistula is at or below the level of the carina [5]. If the placement of a double-lumen tube is not possible, intentional intubation of the right or left main-stem bronchus is an appropriate secondary option. If dual lung ventilation is necessary, or adequate endobronchial intubation may be possible, dual endobronchial tubes may be placed, with independent ventilation of each lung provided by separate ventilators [6]. Following airway securement, in order to deliver nutrition and limit soiling of the lungs with the aspirate, a gastrotomy tube can be placed for suctioning and a jejunostomy tube can be used for enteral nutrition supplementation [1]. The methods described are all used to help optimize the patient preoperatively, and most surgical interventions, which are the definitive treatment of TEFs, are postponed until the patient's clinical condition is stabilized.

The case described above was unique due to the size and location of the TEF with the opening of the fistula being nearly as large as the left main-stem bronchus. With the location being essentially at the level of the carina on the left side, few viable options were available for the hospital team to manage this airway. A single-lumen ETT above the level of the lesion preferentially shunts air to the fistula and leads to the rapid decompensation of respiratory status. Given no prior evaluation of the tracheobronchial tree, there was no initial indication or awareness of the underlying etiology. Selective ventilation and isolation of the right lung, with minimization of left lung soiling, would have been most optimal, but in this case, we had no ready access to this device. A left-sided double-lumen tube would have potentially enlarged the fistula further, and it was unlikely that the endobronchial balloon could be inflated and secured beyond the point of the fistula.

Management algorithms have been developed for acquired tracheoesophageal fistulas, with the location and size of the fistula determining the method of correction. Procedures for correction of the fistula can range from more simple endoscopic procedures to very complex surgeries. Small fistulas may be managed via esophageal stenting using an endoscopic approach or with surgical resection and correction of the defect using a muscular flap [1,2]. Minimal tracheal damage occurs in such a setting. Large fistulas or fistulas with significant tracheal/bronchial damage require more advanced surgical procedures, including, but not limited to, tracheal resection, esophageal diversion, and/or esophageal bypass with colonic interposition [7]. After any of these strategies have been implemented, the patient's trachea is extubated as soon as possible in order to optimize outcomes [2].

## Conclusions

Adult-acquired tracheoesophageal fistulas are rare and must be recognized early in order to decrease the consequences of lung soiling and pulmonary complications. Without early recognition, patients can decompensate quickly leaving few options for treatment. Early temporizing methods, securing an airway, and limiting soiling of the lungs prevent acute decompensation and allow time to optimize a patient’s clinical status until a more definitive surgical solution can be achieved. In community hospitals that do not have access to advanced GI services or specialized surgical teams, damage control and optimizing respiratory status are the only ways to improve a patient’s overall prognosis.
